# Vitamin D Levels Are Associated with Cardiovascular Disease Events but Not with Cardiovascular Disease or Overall Mortality: A Prospective Population-Based Study

**DOI:** 10.3390/nu15184046

**Published:** 2023-09-18

**Authors:** Pollyanna Patriota, Idris Guessous, Serge Rezzi, Pedro Marques-Vidal

**Affiliations:** 1 Swiss Nutrition and Health Foundation, 1066 Epalinges, Switzerland; serge.rezzi@nutritionhealthfoundation.ch; 2 Division of Primary Care Medicine, Department of Primary Care Medicine, Geneva University Hospitals, 1205 Geneva, Switzerland; idris.guessous@hcuge.ch; 3 Department of Medicine, Internal Medicine, Lausanne University Hospital and University of Lausanne, 46 rue du Bugnon, 1011 Lausanne, Switzerland; pedro-manuel.marques-vidal@chuv.ch

**Keywords:** vitamin D, all-cause mortality, prospective study, cardiovascular disease

## Abstract

(1) Background: A recent review concluded that there was no strong evidence for beneficial vitamin D effects on cardiovascular disease (CVD) risk, but whether individuals with vitamin D deficiency have a higher risk of CVD should be further studied. (2) Aims: We assessed the association between vitamin D levels and CVD events, CVD mortality, and overall mortality in a prospective population-based study in Lausanne, Switzerland. (3) Methods: A total of 5684 participants (53.6% women, 52.5 ± 10.7 years) were followed for a median of 14.4 years [interquartile range: 10.7–16.6]. Vitamin D blood levels were categorized as normal (≥75 nmol/L or 30 ng/mL), insufficient (50–74 nmol/L or 21–29 ng/mL), and deficient (<50 nmol/L or 20 ng/mL). (4) Results: In total, 568 cardiovascular events, 114 cardiovascular deaths, and 679 deaths occurred during follow-up. After multivariate analysis, vitamin D levels were negatively associated with CVD events: hazard ratio and (95% confidence interval) for a 10 nmol/L increase: 0.96 (0.92–0.99). However, no association was found for CVD [0.93 (0.84–1.04)] and overall mortality [0.98 (0.94–1.02)]. No associations were found between vitamin D categories and CVD events, 0.93 (0.71–1.22) and 1.14 (0.87–1.49); CVD deaths, 0.78 (0.41–1.50) and 1.10 (0.57–2.12); and overall mortality, 1.10 (0.82–1.48); and 1.17 (0.87–1.58) for insufficiency and deficiency, respectively. After excluding participants taking vitamin D supplements, similar results were obtained. (5) Conclusion: In this prospective population-based study, vitamin D levels were inversely associated with CVD events but not with CVD or overall mortality.

## 1. Introduction

Low vitamin D status has been associated with increased mortality in some populations [1,2]. A meta-analysis of 33 prospective studies on half a million participants found an inverse association between vitamin D concentrations and incident coronary heart disease, stroke, and all-cause mortality among people with low vitamin D levels [3]. Meta-analyses of randomized controlled trials analyzing the effect of vitamin D supplementation have shown small decreases in all-cause mortality among specific groups, such as the elderly and people with cancer, diabetes, or COVID-19 [2,4,5]. For instance, in patients with vitamin D deficiency and no prior history of myocardial infarction, treatment with vitamin D was associated with a significantly lower risk of all-cause mortality [6]. Finally, a recent meta-analysis of randomized controlled trials suggested that vitamin D supplementation could decrease all-cause mortality but not cardiovascular disease (CVD) morbidity and mortality [7]. Still, several limitations of the previous studies that assess the association between vitamin D and mortality have been put forward, such as heterogeneous dosing methods of vitamin D, genetic variation in vitamin D metabolism-related genes, participant diversity, and short follow-up periods [1,2]. A recent narrative review concluded that there was no strong evidence for beneficial vitamin D effects on CVD risk, neither in the general population nor in high-risk groups, but that it should be further studied whether individuals with vitamin D deficiency or a combination of low vitamin D status with specific gene variants have a higher risk of CVD or not [8].

Hence, in this study, we assessed the association between vitamin D levels and cardiovascular events, cardiovascular mortality, and overall mortality, in an apparently healthy, population-based sample living in Lausanne, Switzerland. Our hypothesis was that participants with low vitamin D levels would have a higher incidence of CVD events.

## 2. Participants and Methods

### 2.1. Study Design

The CoLaus (Cohorte Lausannoise) study is a population-based prospective study assessing the clinical, biological, and genetic determinants of cardiovascular disease aged 35 to 75 years at baseline, living in the city of Lausanne, Switzerland. In each survey, participants answered questionnaires and underwent a clinical examination, and blood samples were drawn for analyses. Recruitment began in June 2003, ended in May 2006, and included 6733 participants. The first follow-up was performed between April 2009 and September 2012 (N = 5064 participants), the second follow-up between May 2014 and April 2017 (N = 4881), and the third follow-up between April 2018 and May 2021 (N = 3751). Median follow-up time was 5.4 (average 5.6, range 4.5–8.8) years for the first follow-up, 10.7 (average 10.9, range 8.8–13.6) years for the second follow-up, and 14.5 (average 14.6, range 13.2–17.3) for the third follow-up.

### 2.2. Vitamin D Levels

Blood samples for vitamin D measurement were collected when the participant attended the baseline (2003–2006) visit. Vitamin D was assessed at baseline via an ultra-HPLC tandem-MS system. The calibrators, 3Plus1 Multilevel Serum Calibrator Set 25-OH-Vitamin D3/D2 (ChromoSystems), were standardized against the National Institute of Standards and Technology 972 reference material. The between-day CV% was 4.6% at 40 nmol/L [9]. Vitamin D levels were further categorized as normal (≥75 nmol/L or 30 ng/mL), insufficient (50 to 74 nmol/L or 21 to 29 ng/mL), and deficient (<50 nmol/L or 20 ng/mL) [10].

### 2.3. Death and Cardiovascular Events

During the follow-up period, first incident CVD events and deaths were prospectively collected and independently adjudicated according to established recommendations and similar definitions detailed elsewhere [11].

### 2.4. Other Covariates

Educational level was categorized into high (university), middle (high school), and low (apprenticeship or mandatory). Nationality was categorized as being born in Switzerland or not. Marital status was categorized as living with or without a partner. Smoking status was self-reported and categorized as never, former, and current. No information regarding dietary intake or sun exposure was collected.

Body weight and height were measured with participants barefoot and in light indoor clothes. Body weight was measured in kilograms to the nearest 100 g using a Seca^®^ scale (Hamburg, Germany). Height was measured to the nearest 5 mm using a Seca^®^ (Hamburg, Germany) height gauge. Body mass index (BMI) was calculated and categorized as normal (<25 kg/m^2^), overweight ≥25 and <30 kg/m^2^) and obese ≥30 kg/m^2^).

Blood pressure (BP) was measured using an Omron^®^ HEM-907 automated oscillometric sphygmomanometer after at least a 10-minute rest in a seated position, and the average of the last two measurements was used. Hypertension was defined using an SBP ≥ 140 mm Hg or a DBP ≥ 90 mm Hg or presence of antihypertensive drug treatment.

Biological assays were performed by the CHUV Clinical Laboratory on fresh blood samples within 2 hours of blood collection. Total cholesterol was assessed via CHOD-PAP (maximum inter and intra-batch CVs: 1.6–1.7%); HDL-cholesterol was assessed using CHOD-PAP + PEG + cyclodextrin (maximum inter and intra-batch CVs: 3.6–0.9%); glucose via glucose dehydrogenase (maximum inter and intra-batch CVs: 2.1–1.0%). Diabetes was defined as fasting plasma glucose ≥ 7 mmol/L or presence of antidiabetic drug treatment.

The 10-year risk of developing CVD was computed using SCORE [12] or SCORE-OP [13] risk equations for low-risk countries according to the participant’s age.

Vitamin D and multivitamin supplements were extracted from the list of medicines reported by the participants.

### 2.5. Exclusion Criteria

Participants were excluded if they (1) lacked vitamin D data, (2) presented with CVD at baseline, (3) had no follow-up, and (4) had any missing covariate.

### 2.6. Statistical Analysis

Statistical analysis was conducted using Stata version 16.1 for Windows (Stata corp., College Station, TX, USA). Descriptive statistics were presented as number of participants (percentage) for categorical variables or as average ± standard deviation for continuous variables. Between-group comparisons were performed using chi-square or Fisher’s exact test for categorical variables and analysis of variance for continuous variables.

Bivariate associations between vitamin D categories and the outcomes of interest were assessed using Kaplan–Meyer curves. Multivariate analyses were conducted using Cox model for cardiovascular disease and overall mortality, and Fine–Gray competing risk model for cardiovascular disease mortality, using non-cardiac mortality as competing event. The effects of vitamin D categories and continuous vitamin D levels (per 10 nmol/L increase) were studied. Several models were considered: model 1 adjusted for calendar month of blood draw, age (continuous), sex (male, female), nationality (Swiss, other), education (low, middle, high), marital status (with a partner, without a partner), hypertension (yes, no), diabetes (yes, no), total and HDL cholesterol (continuous), body mass index categories (normal, overweight, obese) and smoking categories (never, former, current); model 2 adjusted for SCORE2 categories (low, intermediate, high), calendar month of blood draw, nationality (Swiss, other), education (low, middle, high), and marital status (with a partner, without a partner); and model 3 adjusted for risk of CVD according to SCORE2 (continuous), calendar month of blood draw, nationality (Swiss, other), education (low, middle, high), and marital status (with a partner, without a partner). The same models were used in a sensitivity analysis that excluded participants taking any supplements susceptible of containing vitamin D. Finally, we conducted dose–response analyses assessing the shape of association between vitamin D levels and outcomes (CVD events and overall mortality) via fractional polynomials, adjusting for the same variables as model 3.

Statistical significance was considered for a two-sided test with *p* < 0.05.

## 3. Results

### 3.1. Characteristics of Participants

Of the initial 6733 participants, 5684 (84.4%) were included in the analyses. The reasons for exclusion are summarized in Supplementary Figure S1, and the characteristics of included and excluded participants are summarized in Supplementary Table S1. Included participants were more frequently women, born in Switzerland, of higher educational level, and presented less frequently with obesity, hypertension, diabetes, and vitamin D deficiency.

Mean (±SD) vitamin D levels were 51 ± 24 nmol/L. Over half of the sample (56.8%) presented with vitamin D deficiency, one-third (31.0%) with vitamin D insufficiency, and 12.2% with normal vitamin D levels. The characteristics of included participants according to vitamin D categories are summarized in Table 1. Participants with vitamin D deficiency were younger, less frequently women and born in Switzerland, more frequently current smokers, and presenting with obesity, hypertension, diabetes, and a very high CVD risk.

### 3.2. Association between Vitamin D Categories, Vitamin D Levels, Cardiovascular Events and Mortality, and Overall Mortality

During a median of 14.4 years [interquartile range: 10.7–16.6] of follow-up, 568 cardiovascular events, 114 cardiovascular deaths, and 679 overall deaths occurred. The associations between vitamin D categories and vitamin D levels with cardiovascular events and mortality are indicated in Table 2. No significant association was found between vitamin D categories and CVD events, CVD, or overall mortality. When vitamin D levels were considered as a continuous variable, significant inverse associations were found with CVD events, and similar albeit non-significant associations were found for CVD and overall mortality (Table 2).

**Figure 1:**
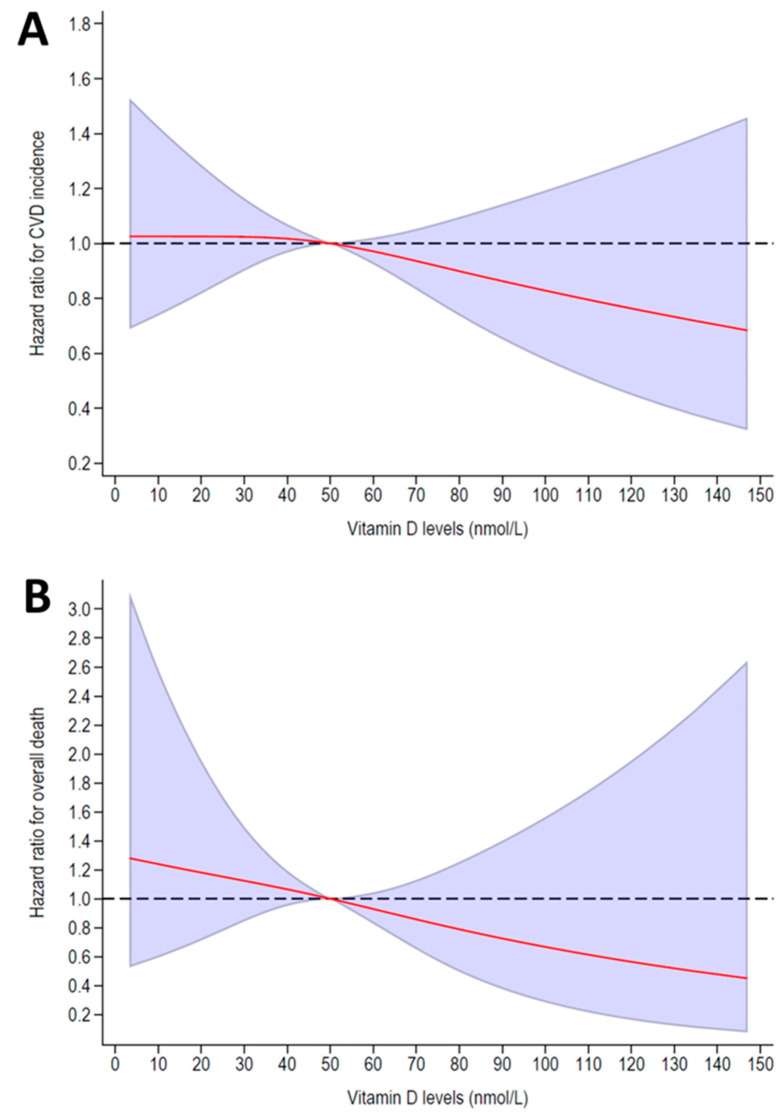
Associations between vitamin D levels and cardiovascular events (**panel A**) and overall mortality (**panel B**). The reference value is 50 nmol/L. The red line corresponds to the hazard ratio and the colored areas represent the 95% confidence intervals for the dose–response curves.

Associations for CVD events showed a null association for vitamin D concentrations below 50 ng/mL, and a trend towards a negative association for vitamin D concentrations above 50 ng/mL (Figure 1, panel A). For overall mortality, a non-significant, negative association was found (Figure 1, panel B).

The results after excluding 712 participants taking vitamin supplements are summarized in Table 3. No significant association was found between vitamin D categories and levels with any of the considered outcomes, while the negative associations between vitamin D levels and CVD events were replicated, and the negative associations between vitamin D levels and CVD or overall mortality reached statistical significance in some models (Table 3).

## 4. Discussion

In this study, we found an inverse association between vitamin D levels and CVD events, but not with CVD or overall mortality. No association was found between vitamin D insufficiency or deficiency and cardiovascular outcomes or overall mortality.

### 4.1. Characteristics of Participants

Over half of the sample (56.8%) presented with vitamin D deficiency, one-third (31.0%) with vitamin D insufficiency, and 12.2% with normal vitamin D levels. Our findings are consistent with those of other studies [3,14]. For instance, among the 26 population-based cohorts included in a meta-analysis, 24 had mean vitamin D levels below 75 nmol/L, and 7 below 50 nmol/L [3]. Overall, our results indicate that the prevalence of vitamin D insufficiency and deficiency is high in the general population of Lausanne.

### 4.2. Association between Vitamin D Levels, Vitamin D Categories, Cardiovascular Events and Mortality, and Overall Mortality

Vitamin D levels were inversely associated with CVD events and to a lesser degree to CVD or overall mortality. Our results partly replicate those of a Chinese study, where increased serum vitamin D levels were associated with lower CVD and overall mortality [2], and those of a study including 33 prospective studies, where increased vitamin D levels were associated with lower CVD and overall mortality among people with vitamin D levels below 40 nmol/L but not among people with higher vitamin D levels [3]. The lack of association between vitamin D and mortality agrees with a recent meta-analysis of randomized controlled trials, which showed that vitamin D supplementation could reduce CVD events but not CVD or overall mortality [7]. Surprisingly, the dose–response associations between vitamin D levels and CVD events or overall mortality were the mirror of those reported in the previous study [3], with a null association being found for vitamin D levels below 50 nmol/L and a negative association for values above this threshold. Still, the dose–response curves observed in this study were comparable to those of EPIC-CVD, as reported elsewhere [3]. Overall, our results suggest that increasing vitamin D levels might be associated with lower incidence of CVD events, while the association with mortality could not be established,

No associations were found between vitamin D categories and all outcomes studied. A study conducted in the UK Biobank concluded that genetically determined vitamin D deficiency was associated with increased CVD and overall mortality [1], while no such relationship was found in another study also using genetically determined vitamin D levels [3]. Indeed, a recent narrative review noted the need to experiment with whether vitamin D supplementation could benefit people with vitamin D deficiency [8]. It is also possible that the observed association between vitamin D and CVD outcomes may be due to people with extreme deficiencies, as per the dose–response graphs published previously [3]. Overall, it seems that categorizing vitamin D levels into insufficiency and deficiency does not add further information in assessing the associations between vitamin D and CVD outcomes.

### 4.3. Possible Mechanisms

Several mechanisms by which vitamin D influences CVD have been proposed. Vitamin D supplementation has been suggested to decrease total and LDL cholesterol and triglyceride levels in one study [15], total cholesterol and triglycerides among the elderly in another [16], only triglyceride levels in postmenopausal women in a third [17], and to exert no effect in people with metabolic syndrome in a fourth study [18]. Further, while the previously mentioned studies failed to find any effect of vitamin D supplementation on HDL cholesterol levels, yet another study reported an increase in HDL cholesterol after vitamin D supplementation among people with cardiovascular disease [19]. Vitamin D supplementation does not decrease BP levels, although a beneficial effect among vitamin D deficient, hypertensive patients was suggested [20,21]. Vitamin D levels are inversely associated with the risk of diabetes [22], and vitamin D supplementation leads to improvements in glucose and insulin levels [19] and prevents evolution to diabetes among people with prediabetes [23]. Finally, whether vitamin D represents a bystander-only indicator or a causal factor amenable to treatment in CVD is still debated. Similarly, whether supplementing patients at high risk of CVD and with vitamin D insufficiency is beneficial or not regarding CVD outcomes, warrants further investigation [24,25]. Also, mechanistic studies, namely regarding the biochemical pathways influenced by vitamin D and acting on CVD risk, should be further studied.

Overall, the available information suggests a beneficial effect of vitamin D supplementation on cardiovascular risk factors, but results differ according to the population studied. This effect could explain the association between vitamin D levels and CVD events.

### 4.4. Strengths and Limitations

The main strengths of this study are the assessment of blood vitamin D levels, its prospective setting, and adjusting for a large set of covariates. In addition, the findings replicate those of other studies conducted in other countries.

This study also has several limitations. First, the number of events was relatively small, leading to a low statistical power. Hence, it is possible that the non-significant, negative associations between vitamin D levels and CVD or overall mortality would become significant had we relied on a larger sample. Second, it was not possible to assess the dietary intake of vitamin D in the absence of dietary assessment. Still, as a significant amount of vitamin D (80–90%) comes from sun exposure [26], and as we adjusted for a month of blood drawing, we believe that the omission of the food source should not represent a major bias. Third, blood vitamin D levels were measured only once at the beginning of this study, and changes were not assessed during the follow-up. Hence, it is possible that the vitamin D condition of some participants might have changed.

## 5. Conclusions

In this prospective, population-based study, vitamin D levels were inversely associated with CVD events but not with CVD or overall mortality. The inverse association was not confirmed when vitamin D levels were categorized into normal, insufficient, or deficient.

## Figures and Tables

**Table 1:** Characteristics of participants according to vitamin D categories, CoLaus study, Lausanne, Switzerland.

	Deficient	Insufficient	Normal	*p*-Value
N (% of total)	3229 (56.8)	1760 (31.0)	695 (12.2)	
Women (%)	1673 (51.8)	984 (55.9)	392 (56.4)	0.006
Age (years)	51.9 ± 10.6	52.9 ± 10.7	54.4 ± 10.8	<0.001
Born in Switzerland (%)	1866 (57.8)	1191 (67.7)	495 (71.2)	<0.001
Marital status: with a partner (%)	2152 (66.7)	1202 (68.3)	454 (65.3)	0.289
Educational level (%)				0.079
High	647 (20.0)	368 (20.9)	138 (19.9)	
Middle	774 (24.0)	453 (25.7)	198 (28.5)	
Low	1808 (56.0)	939 (53.4)	359 (51.7)	
Smoking categories (%)				0.004
Never	1300 (40.3)	733 (41.7)	288 (41.4)	
Former	1020 (31.6)	584 (33.2)	255 (36.7)	
Current	909 (28.2)	443 (25.2)	152 (21.9)	
Body mass index (kg/m^2^)	26.1 ± 4.6	25.2 ± 4.1	24.2 ± 3.7	<0.001
BMI categories (%)				<0.001
Normal	1456 (45.1)	939 (53.4)	435 (62.6)	
Overweight	1229 (38.1)	619 (35.2)	211 (30.4)	
Obese	544 (16.9)	202 (11.5)	49 (7.1)	
Hypertension (%)	1195 (37.0)	557 (31.7)	231 (33.2)	<0.001
Diabetes (%)	238 (7.4)	75 (4.3)	21 (3.0)	<0.001
SCORE risk categories (%)				<0.001
Low-intermediate	1898 (59.3)	1114 (64.1)	434 (63.1)	
High	885 (27.6)	420 (24.2)	198 (28.8)	
Very high	419 (13.1)	203 (11.7)	56 (8.1)	

Results are expressed as number of participants (column percentage) for categorical variables and mean ± standard deviation for continuous variables. Between-group comparisons performed using chi-square for categorical variables and via analysis of variance for continuous variables.

**Table 2:** Association between vitamin D categories or vitamin D levels and cardiovascular disease, cardiovascular mortality, and overall mortality, CoLaus study, Lausanne, Switzerland.

	Deficient	*p*-Value	Insufficient	*p*-Value	Normal	*p*-Value for Trend	Per 10 nmol/L Increase	*p*-Value
N	3229		1760		695			
Cardiovascular events								
Unadjusted	1.22 (0.93–1.60)	0.156	1.12 (0.84–1.50)	0.440	1 (ref.)	0.156	0.92 (0.89–0.95)	<0.001
Adjusted, model 1	1.26 (0.96–1.65)	0.097	1.04 (0.79–1.37)	0.773	1 (ref.)	0.097	0.93 (0.89–0.96)	<0.001
Adjusted, model 2	1.08 (0.83–1.42)	0.562	0.91 (0.69–1.20)	0.500	1 (ref.)	0.562	0.94 (0.90–0.98)	0.003
Adjusted, model 3	1.14 (0.87–1.49)	0.349	0.93 (0.71–1.22)	0.602	1 (ref.)	0.349	0.96 (0.92–0.99)	0.030
Cardiovascular mortality								
Unadjusted	1.00 (0.57–1.74)	0.994	0.77 (0.42–1.44)	0.418	1 (ref.)	0.994	0.93 (0.85–1.02)	0.118
Adjusted, model 1	1.16 (0.60–2.22)	0.661	0.90 (0.47–1.73)	0.751	1 (ref.)	0.661	0.92 (0.83–1.02)	0.097
Adjusted, model 2	1.07 (0.55–2.07)	0.845	0.84 (0.44–1.63)	0.608	1 (ref.)	0.845	0.92 (0.83–1.02)	0.129
Adjusted, model 3	1.10 (0.57–2.12)	0.784	0.78 (0.41–1.50)	0.461	1 (ref.)	0.784	0.93 (0.84–1.04)	0.229
Overall mortality								
Unadjusted	1.22 (0.96–1.56)	0.111	0.93 (0.71–1.22)	0.620	1 (ref.)	0.111	0.96 (0.92–0.99)	0.028
Adjusted, model 1	1.25 (0.93–1.69)	0.140	1.18 (0.87–1.59)	0.281	1 (ref.)	0.140	0.96 (0.92–1.00)	0.062
Adjusted, model 2	1.12 (0.83–1.50)	0.466	1.07 (0.79–1.44)	0.654	1 (ref.)	0.466	0.97 (0.93–1.01)	0.188
Adjusted, model 3	1.17 (0.87–1.58)	0.288	1.10 (0.82–1.48)	0.542	1 (ref.)	0.288	0.98 (0.94–1.02)	0.302

Results are expressed as hazard rate (95% confidence interval) for cardiovascular events and overall mortality and a sub-hazard rate (95% confidence interval) for cardiovascular mortality. Statistical analysis using Cox model for cardiovascular events and overall mortality, and Fine–Gray competing risk model for cardiovascular disease mortality, using non-cardiac mortality as competing event. Model 1 adjusted for calendar month of blood draw, age (continuous), sex (male, female), nationality (Swiss, other), education (low, middle, high), marital status (with a partner, without a partner), hypertension (yes, no), diabetes (yes, no), total and HDL cholesterol (continuous), body mass index categories (normal, overweight, obese) and smoking categories (never, former, current). Model 2 adjusted for SCORE2 categories (low, intermediate, high), calendar month of blood draw, nationality (Swiss, other), education (low, middle, high), and marital status (with a partner, without a partner). Model 3 adjusted for risk of CVD according to SCORE2 (continuous), calendar month of blood draw, nationality (Swiss, other), education (low, middle, high), and marital status (with a partner, without a partner).

**Table 3:** Association between vitamin D categories, vitamin D levels and cardiovascular disease, cardiovascular mortality, and overall mortality, excluding participants consuming vitamin supplements, CoLaus study, Lausanne, Switzerland.

	Deficient	*p*-Value	Insufficient	*p*-Value	Normal	*p*-Value for Trend	Per 10 nmol/L Increase	*p*-Value
N	2917		1494		561			
Cardiovascular events								
Unadjusted	1.28 (0.94–1.74)	0.113	1.19 (0.86–1.66)	0.289	1 (ref.)	0.113	0.92 (0.88–0.95)	<0.001
Adjusted, model 1	1.24 (0.92–1.67)	0.166	1.02 (0.75–1.39)	0.903	1 (ref.)	0.166	0.93 (0.89–0.97)	0.001
Adjusted, model 2	1.06 (0.79–1.43)	0.707	0.85 (0.62–1.16)	0.301	1 (ref.)	0.707	0.93 (0.89–0.98)	0.003
Adjusted, model 3	1.12 (0.83–1.51)	0.448	0.89 (0.66–1.21)	0.452	1 (ref.)	0.448	0.95 (0.91–0.99)	0.027
Cardiovascular mortality								
Unadjusted	1.02 (0.55–1.90)	0.943	0.68 (0.34–1.37)	0.280	1 (ref.)	0.943	0.90 (0.81–1.00)	0.053
Adjusted, model 1	1.23 (0.59–2.58)	0.576	0.82 (0.38–1.75)	0.610	1 (ref.)	0.576	0.88 (0.79–0.99)	0.042
Adjusted, model 2	1.07 (0.52–2.19)	0.847	0.70 (0.34–1.44)	0.329	1 (ref.)	0.847	0.89 (0.78–1.00)	0.053
Adjusted, model 3	1.20 (0.58–2.51)	0.624	0.71 (0.34–1.49)	0.368	1 (ref.)	0.624	0.90 (0.79–1.02)	0.095
Overall mortality								
Unadjusted	1.21 (0.93–1.59)	0.161	0.89 (0.66–1.20)	0.456	1 (ref.)	0.161	0.95 (0.91–0.99)	0.022
Adjusted, model 1	1.41 (1.01–1.98)	0.045	1.33 (0.95–1.86)	0.097	1 (ref.)	0.045	0.95 (0.90–0.99)	0.023
Adjusted, model 2	1.21 (0.87–1.69)	0.259	1.14 (0.82–1.60)	0.433	1 (ref.)	0.259	0.96 (0.91–1.00)	0.066
Adjusted, model 3	1.29 (0.92–1.80)	0.138	1.20 (0.86–1.68)	0.275	1 (ref.)	0.138	0.97 (0.92–1.01)	0.146

Results are expressed as hazard rate (95% confidence interval) for cardiovascular events and overall mortality and a sub-hazard rate (95% confidence interval) for cardiovascular mortality. Statistical analysis using Cox model for cardiovascular events and overall mortality, and Fine–Gray competing risk model for cardiovascular disease mortality, using non-cardiac mortality as competing event. Model 1 adjusted for calendar month of blood draw, age (continuous), sex (male, female), nationality (Swiss, other), education (low, middle, high), marital status (with a partner, without a partner), hypertension (yes, no), diabetes (yes, no), total and HDL cholesterol (continuous), body mass index categories (normal, overweight, obese) and smoking categories (never, former, current). Model 2 adjusted for SCORE2 categories (low, intermediate, high), calendar month of blood draw, nationality (Swiss, other), education (low, middle, high), and marital status (with a partner, without a partner). Model 3 adjusted for risk of CVD according to SCORE2 (continuous), calendar month of blood draw, nationality (Swiss, other), education (low, middle, high), and marital status (with a partner, without a partner).

## Data Availability

The data of the CoLaus|PsyCoLaus study used in this article cannot be fully shared as they contain potentially sensitive personal information on participants. According to the Ethics Committee for Research of the Canton of Vaud, sharing these data would be a violation of the Swiss legislation with respect to privacy protection. However, coded individual-level data that do not allow researchers to identify participants are available upon request to researchers who meet the criteria for data sharing of the CoLaus|PsyCoLaus Datacenter (CHUV, Lausanne, Switzerland). Any researcher affiliated with a public or private research institution who complies with the CoLaus|PsyCoLaus standards can submit a research application to research.colaus@chuv.ch or research.psycolaus@chuv.ch. Proposals requiring baseline data only will be evaluated using the baseline (local) Scientific Committee (SC) of the CoLaus and PsyCoLaus studies. Proposals requiring follow-up data will be evaluated by the follow-up (multicentric) SC of the CoLaus|PsyCoLaus cohort study. Detailed instructions for gaining access to the CoLaus|PsyCoLaus data used in this study are available at www.colaus-psycolaus.ch/professionals/how-to-collaborate/.

## Supplementary Materials

The following supporting information can be downloaded at: https://www.mdpi.com/article/10.3390/nu15184046/s1, Figure S1: Participant Selection; Table S1: characteristics of included and excluded participants, CoLaus study, Lausanne, Switzerland.

## References

[1] Sutherland J.P., Zhou A., Hyppönen E. (2022). Vitamin D Deficiency Increases Mortality Risk in the UK Biobank: A nonlinear Mendelian randomization study. Ann. Intern. Med..

[2] Wan Z., Guo J., Pan A., Chen C., Liu L., Liu G. (2020). Association of Serum 25-Hydroxyvitamin D Concentrations With All-Cause and Cause-Specific Mortality Among Individuals With Diabetes. Diabetes Care.

[3] Sofianopoulou E., Kaptoge S.K., Afzal S., Jiang T., Gill D., Gundersen T.E., Bolton T.R., Allara E., Arnold M.G., Mason A.M. (2021). Estimating dose-response relationships for vitamin D with coronary heart disease, stroke, and all-cause mortality: Observational and Mendelian randomisation analyses. Lancet Diabetes Endocrinol..

[4] Varikasuvu S.R., Thangappazham B., Vykunta A., Duggina P., Manne M., Raj H., Aloori S. (2022). COVID-19 and vitamin D (Co-VIVID study): A systematic review and meta-analysis of randomized controlled trials. Expert Rev. Anti-Infect. Ther..

[5] Zhang Y., Fang F., Tang J., Jia L., Feng Y., Xu P., Faramand A. (2019). Association between vitamin D supplementation and mortality: Systematic review and meta-analysis. BMJ.

[6] Acharya P., Dalia T., Ranka S., Sethi P., Oni O.A., Safarova M.S., Parashara D., Gupta K., Barua R.S. (2021). The Effects of Vitamin D Supplementation and 25-hydroxyvitamin D Levels on The Risk of MI and Mortality. J. Endocr. Soc..

[7] Ruiz-García A., Pallarés-Carratalá V., Turégano-Yedro M., Torres F., Sapena V., Martin-Gorgojo A., Martin-Moreno J.M. (2023). Vitamin D Supplementation and Its Impact on Mortality and Cardiovascular Outcomes: Systematic Review and Meta-Analysis of 80 Randomized Clinical Trials. Nutrients.

[8] Zittermann A., Trummer C., Theiler-Schwetz V., Lerchbaum E., März W., Pilz S. (2021). Vitamin D and Cardiovascular Disease: An Updated Narrative Review. Int. J. Mol. Sci..

[9] Bruce S.J., Rochat B., Béguin A., Pesse B., Guessous I., Boulat O., Henry H. (2012). Analysis and quantification of vitamin D metabolites in serum by ultra-performance liquid chromatography coupled to tandem mass spectrometry and high-resolution mass spectrometry—A method comparison and validation. Rapid Commun. Mass Spectrom..

[10] Holick M.F., Binkley N.C., Bischoff-Ferrari H.A., Gordon C.M., Hanley D.A., Heaney R.P., Murad M.H., Weaver C.M. (2011). Evaluation, Treatment, and Prevention of Vitamin D Deficiency: An Endocrine Society Clinical Practice Guideline. Med. J. Clin. Endocrinol. Metab..

[11] Beuret H., Hausler N., Nanchen D., Méan M., Marques-Vidal P., Vaucher J. (2020). Comparison of Swiss and European risk algorithms for cardiovascular prevention in Switzerland. Eur. J. Prev. Cardiol..

[12] SCORE2 Working Group, ESC Cardiovascular Risk Collaboration (2021). SCORE2 risk prediction algorithms: New models to estimate 10-year risk of cardiovascular disease in Europe. Eur. Heart J..

[13] SCORE2-OP Working Group, ESC Cardiovascular Risk Collaboration (2021). SCORE2-OP risk prediction algorithms: Estimating incident cardiovascular event risk in older persons in four geographical risk regions. Eur. Heart J..

[14] Holick M.F. (2017). The vitamin D deficiency pandemic: Approaches for diagnosis, treatment and prevention. Rev. Endocr. Metab. Disord..

[15] Dibaba D.T. (2019). Effect of vitamin D supplementation on serum lipid profiles: A systematic review and meta-analysis. Nutr. Rev..

[16] Qorbani M., Zarei M., Moradi Y., Appannah G., Djalainia S., Pourrostami K., Ejtahed H.-S., Mahdavi-Gorabi A., Naderali E.K., Khazdouz M. (2022). Effect of vitamin D supplementation on cardiac-metabolic risk factors in elderly: A systematic review and meta-analysis of clinical trials. Diabetol. Metab. Syndr..

[17] Zhang W., Yi J., Liu D., Wang Y., Jamilian P., Gaman M.-A., Prabahar K., Fan J. (2022). The effect of vitamin D on the lipid profile as a risk factor for coronary heart disease in postmenopausal women: A meta-analysis and systematic review of randomized controlled trials. Exp. Gerontol..

[18] AlAnouti F., Abboud M., Papandreou D., Mahboub N., Haidar S., Rizk R. (2020). Effects of Vitamin D Supplementation on Lipid Profile in Adults with the Metabolic Syndrome: A Systematic Review and Meta-Analysis of Randomized Controlled Trials. Nutrients.

[19] Ostadmohammadi V., Milajerdi A., Ghayour-Mobarhan M., Ferns G., Taghizadeh M., Badehnoosh B., Mirzaei H., Asemi Z. (2019). The Effects of Vitamin D Supplementation on Glycemic Control, Lipid Profiles and C-Reactive Protein Among Patients with Cardiovascular Disease: A Systematic Review and Meta-Analysis of Randomized Controlled Trials. Curr. Pharm. Des..

[20] Farapti F., Fadilla C., Yogiswara N., Adriani M. (2020). Effects of vitamin D supplementation on 25(OH)D concentrations and blood pressure in the elderly: A systematic review and meta-analysis. F1000Research.

[21] Mirhosseini N., Vatanparast H., Kimball S.M. (2017). The Association between Serum 25(OH)D Status and Blood Pressure in Participants of a Community-Based Program Taking Vitamin D Supplements. Nutrients.

[22] Mohammadi S., Hajhashemy Z., Saneei P. (2021). Serum vitamin D levels in relation to type-2 diabetes and prediabetes in adults: A systematic review and dose–response meta-analysis of epidemiologic studies. Crit. Rev. Food Sci. Nutr..

[23] Pittas A.G., Kawahara T., Jorde R., Dawson-Hughes B., Vickery E.M., Angellotti E., Nelson J., Trikalinos T.A., Balk E.M. (2023). Vitamin D and Risk for Type 2 Diabetes in People With Prediabetes: A systematic review and meta-analysis of individual participant data from 3 randomized clinical trials. Ann. Intern. Med..

[24] Cosentino N., Campodonico J., Milazzo V., De Metrio M., Brambilla M., Camera M., Marenzi G. (2021). Vitamin D and Cardiovascular Disease: Current Evidence and Future Perspectives. Nutrients.

[25] Luo W., Xu D., Zhang J., Zhou Y., Yang Q., Lv Q., Qu Z. (2022). Low serum 25-hydroxyvitamin D levels are associated with increased cardiovascular morbidity and mortality. Postgrad. Med..

[26] National Health Service Vitamin D. https://www.nhs.uk/conditions/vitamins-and-minerals/vitamin-d/.

