# Supplementary material for: Vitamin D Levels Are Associated with Cardiovascular Disease Events but Not with Cardiovascular Disease or Overall Mortality: A Prospective Population-Based Study
Source: Nutrients. 2023 Sep 18;15(18):4046. doi: 10.3390/nu15184046 (PMC10534692; doi:10.3390/nu15184046)
Supplement: Supplementary file 1 [file nutrients-15-04046-s001.zip › Supplementary Table S1.pdf]

Supplementary information

Supplementary Table S1: characteristics of included and excluded participants, CoLaus study, Lausanne, Switzerland.

	**Included**	**Excluded**	**P-value**
N	5684	1049	
Women (%)	3049 (53.6)	495 (47.2)	<0.001
Age (years)	52.5 ± 10.7	53.2 ± 11	0.067
Born in Switzerland (%)	3552 (62.5)	479 (45.8)	<0.001
Marital status: with a partner (%)	3808 (67.0)	704 (67.5)	0.757
Educational level (%)			<0.001
High	1153 (20.3)	167 (16.1)	
Middle	1425 (25.1)	200 (19.3)	
Low	3106 (54.6)	668 (64.5)	
Smoking categories (%)			0.119
Never	2321 (40.8)	411 (39.4)	
Former	1859 (32.7)	324 (31.1)	
Current	1504 (26.5)	308 (29.5)	
Body mass index (kg/m^2)	25.6 ± 4.4	26.8 ± 4.9	<0.001
BMI categories (%)			<0.001
Normal	2830 (49.8)	407 (38.9)	
Overweight	2059 (36.2)	403 (38.5)	
Obese	795 (14.0)	236 (22.6)	
Hypertension (%)	1983 (34.9)	517 (49.6)	<0.001
Diabetes (%)	334 (5.9)	102 (9.9)	<0.001
SCORE risk categories (%)			<0.001
Low-moderate	3446 (61.2)	495 (48.8)	
High	1503 (26.7)	253 (24.9)	
Very high	678 (12.1)	142 (14.0)	
Previous CVD	-	125 (12.3)	
Vitamin D categories (%)			<0.001
Normal	695 (12.2)	88 (9.1)	
Insufficiency	1760 (31.0)	250 (25.8)	
Deficiency	3229 (56.8)	632 (65.2)	

BMI, body mass index. Results are expressed as number of participants (column percentage) for categorical variables and mean ± standard deviation for continuous variables. Between-group comparisons performed using chi-square for categorical variables and by student's t-test for continuous variables.
