# Supplementary figures and images for: Vitamin D Levels Are Associated with Cardiovascular Disease Events but Not with Cardiovascular Disease or Overall Mortality: A Prospective Population-Based Study
Source: Nutrients. 2023 Sep 18;15(18):4046. doi: 10.3390/nu15184046 (PMC10534692; doi:10.3390/nu15184046)

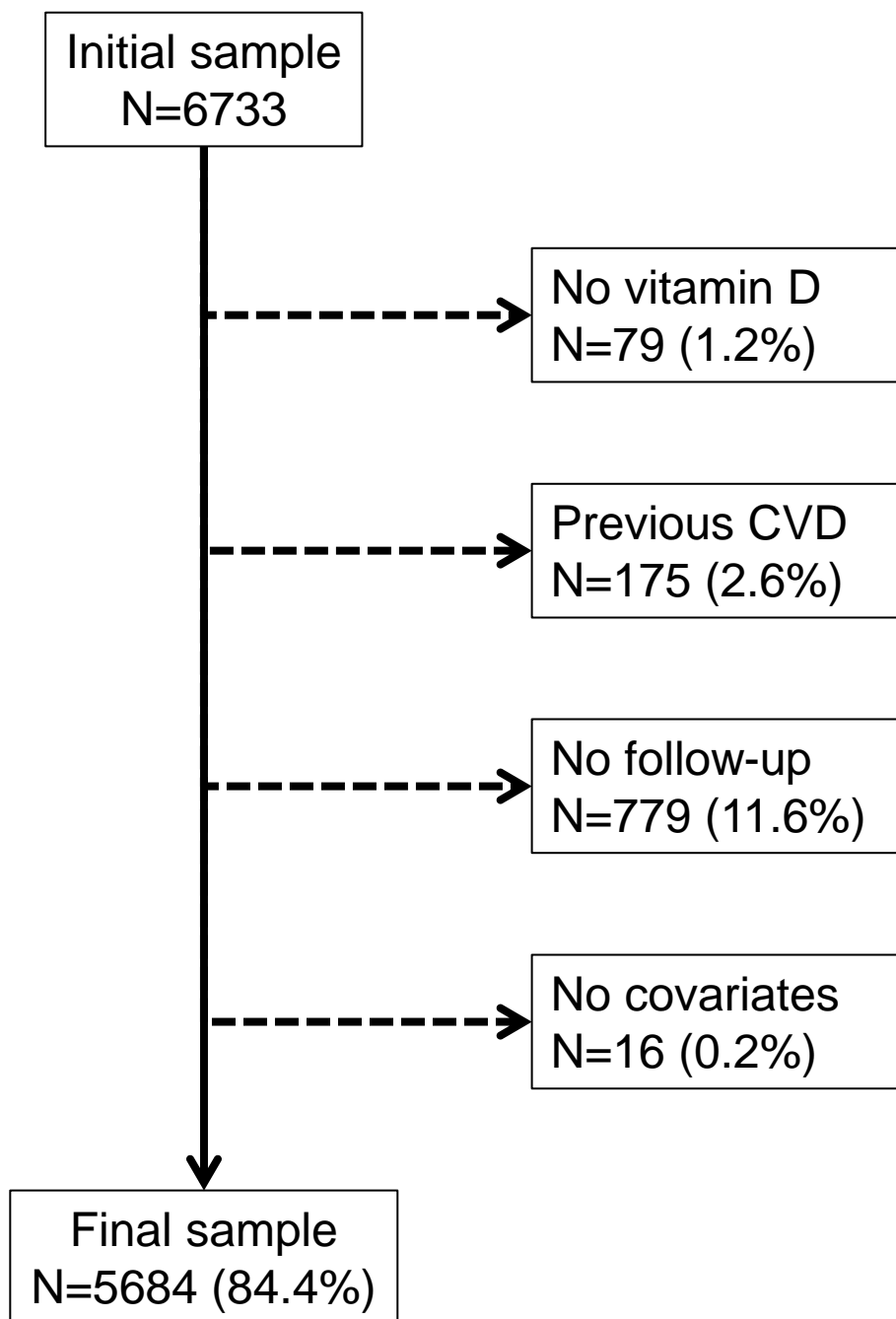

Supplement: Supplementary file 1 [file nutrients-15-04046-s001.zip › Supplementary Figure S1.pdf]
